# Supplementary material for: Abundance of non-conservative microplastics in the upper ocean from 1957 to 2066
Source: Nat Commun. 2019 Jan 24;10:417. doi: 10.1038/s41467-019-08316-9 (PMC6345988; doi:10.1038/s41467-019-08316-9)
Supplement: Supplementary file 1 — Supplementary Information [file 41467_2019_8316_MOESM1_ESM.pdf]

Supplementary Information for

Abundance of non-conservative microplastics in the upper ocean from 1957 to 2066

Isobe, et al.,

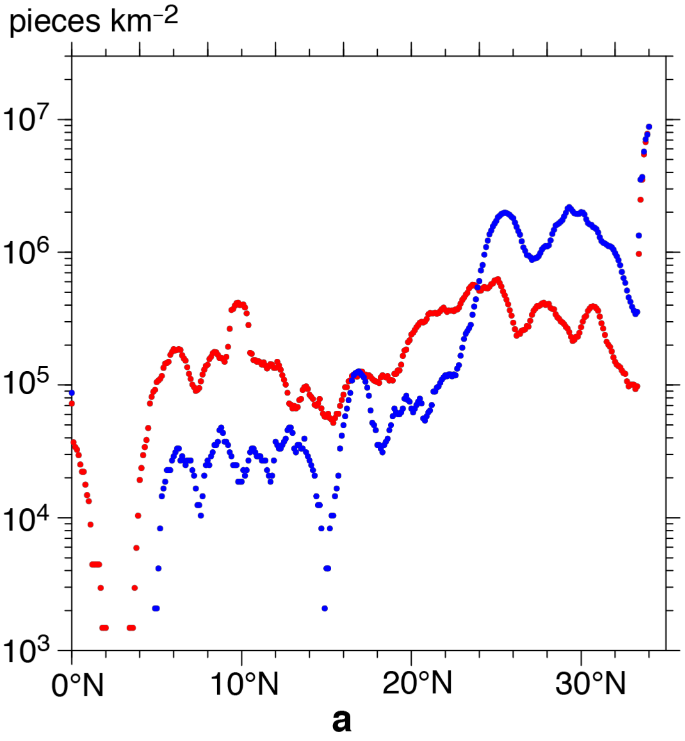

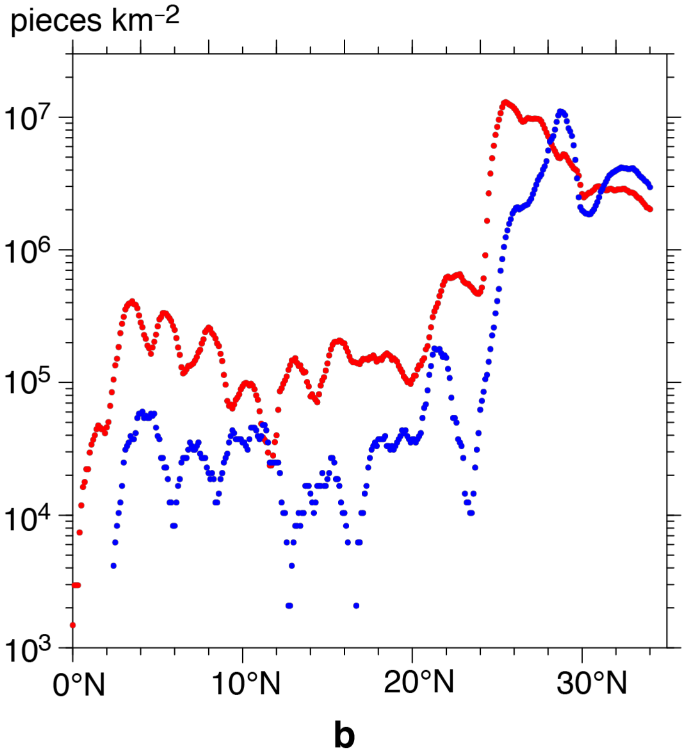

Supplementary Fig. 1. Dependence of particle positions on the Stokes drift. Total particle counts in the model with (red dots) and without (blue dots) the Stokes drift are plotted along 140 (160)°E in the North (South) Pacific (a), and along 180°E (b).

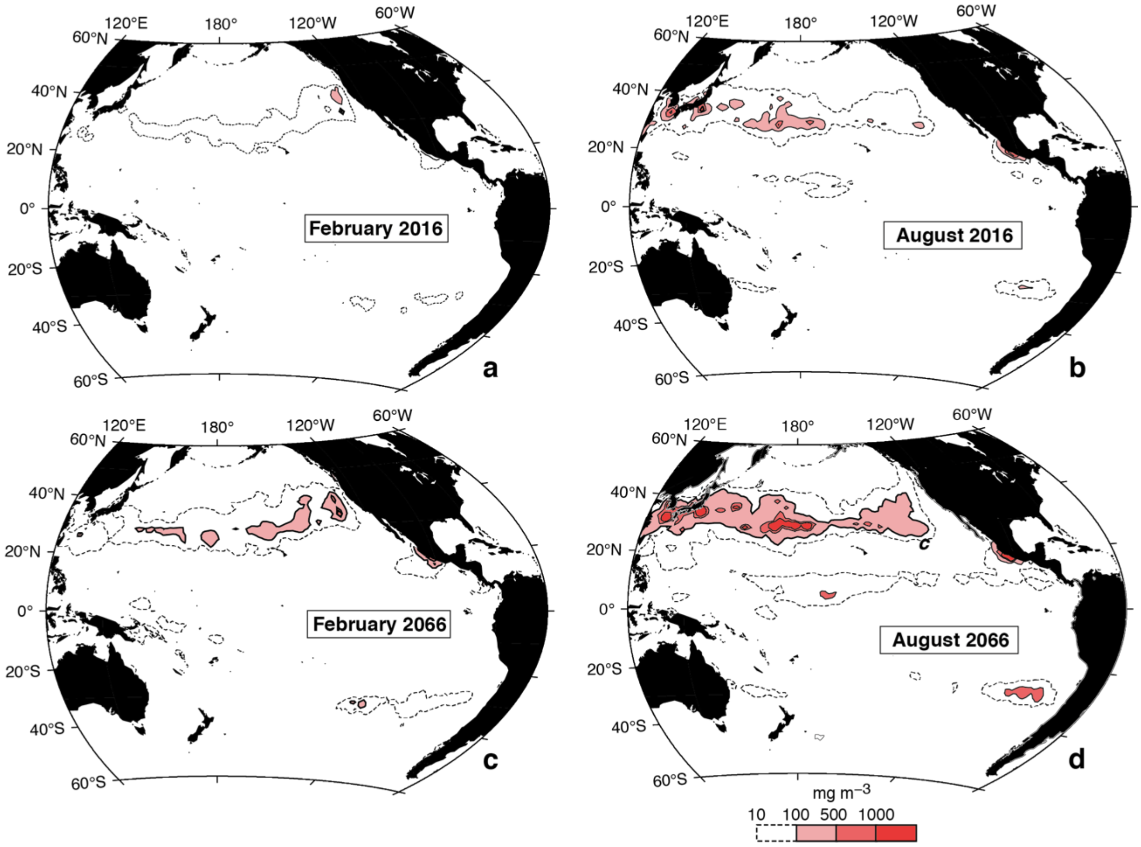

Supplementary Fig. 2. Abundance of microplastics in the present and future. The weight concentrations at the sea surface in the model with fishery-based sources[66] were averaged in February (a) and August (b) in 2016, and February (c) and August (d) in 2066. The weight concentrations are shown by a red stippling in the line with the scale at the bottom of the panel d. The broken curves denote a weight concentration of 10 mg m^{-3}.

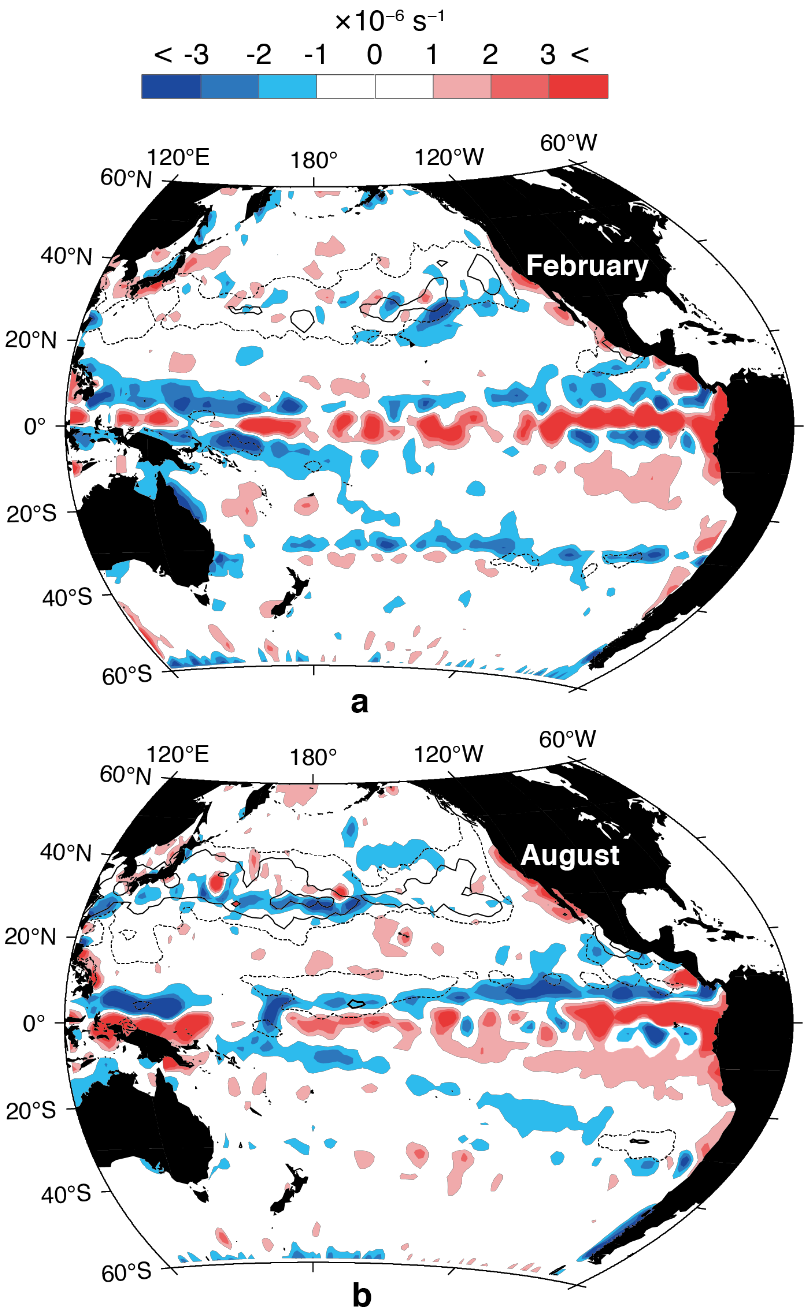

Supplementary Fig. 3. Surface current field used in the numerical model. Maps of divergence of surface currents computed using the HYCOM analysis product and Stokes drift in February (a) and August (b) 2015. Positive (red) and negative (blue) values represent divergence and convergence, respectively; magnitudes are shown in the scale above the panel a. Broken and solid curves indicate modeled weight concentrations of 10, 100, and 1000 mg m^{-3} reproduced in the the same month.

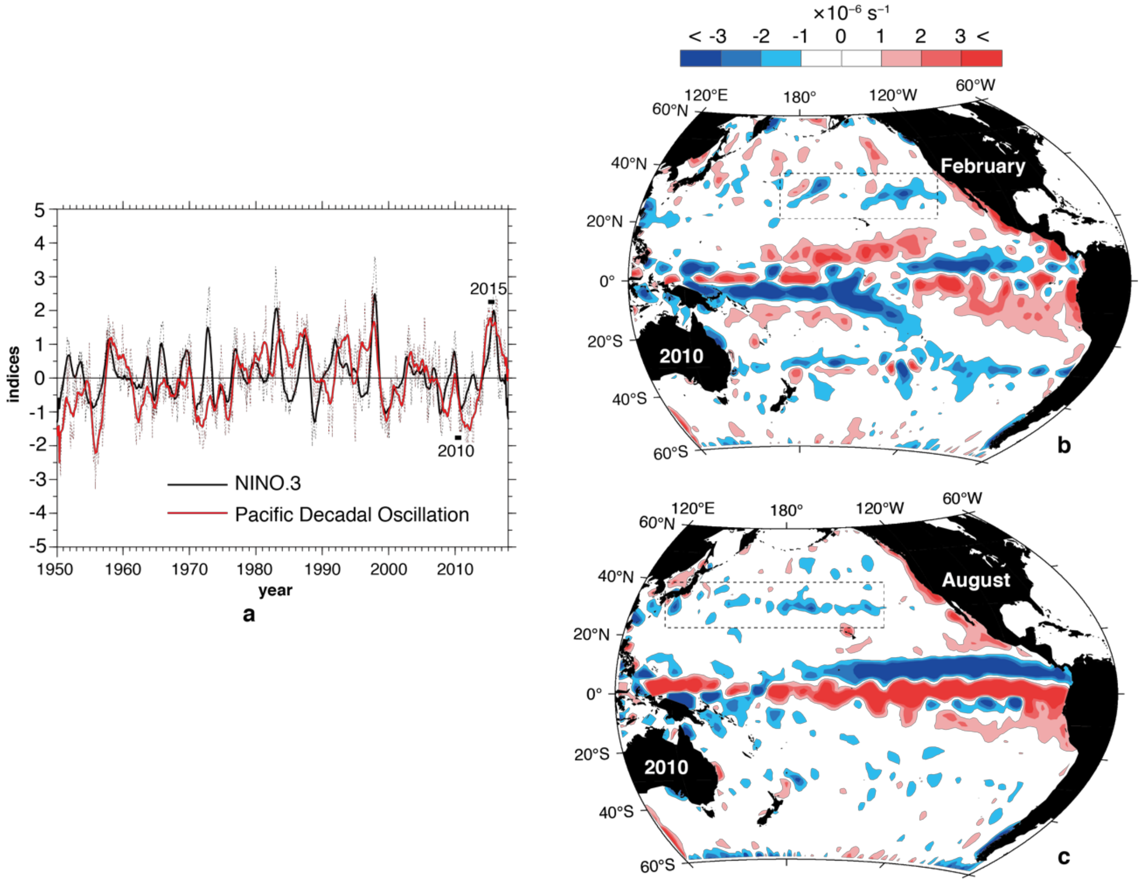

Supplementary Fig. 4. Surface current field used in an additional experiment. The surface currents in 2010 are used to depict the panels b and c, because the Pacific Decadal Oscillation and NINO.3 indices for 2010 were in opposite phase to those in 2015 (a). The monthly Pacific Decadal Oscillation index was downloaded from the Japan Metrological Agency (JMA) website (https://www.data.jma.go.jp/kaiyou/data/shindan/b_1/pdo/pdo.txt). The monthly NINO.3 index (i.e., anomaly of sea surface temperature averaged over 5°S to 5°N and 150°W to 90°W) was also downloaded from the JMA website (https://www.data.jma.go.jp/gmd/cpd/db/elnino/index/nino3idx.html). Thin broken (bold solid) curves indicate monthly averaged data (annually) in the panel a. Areas with intense convergence zones are surrounded by broken lines in the panel b, c.

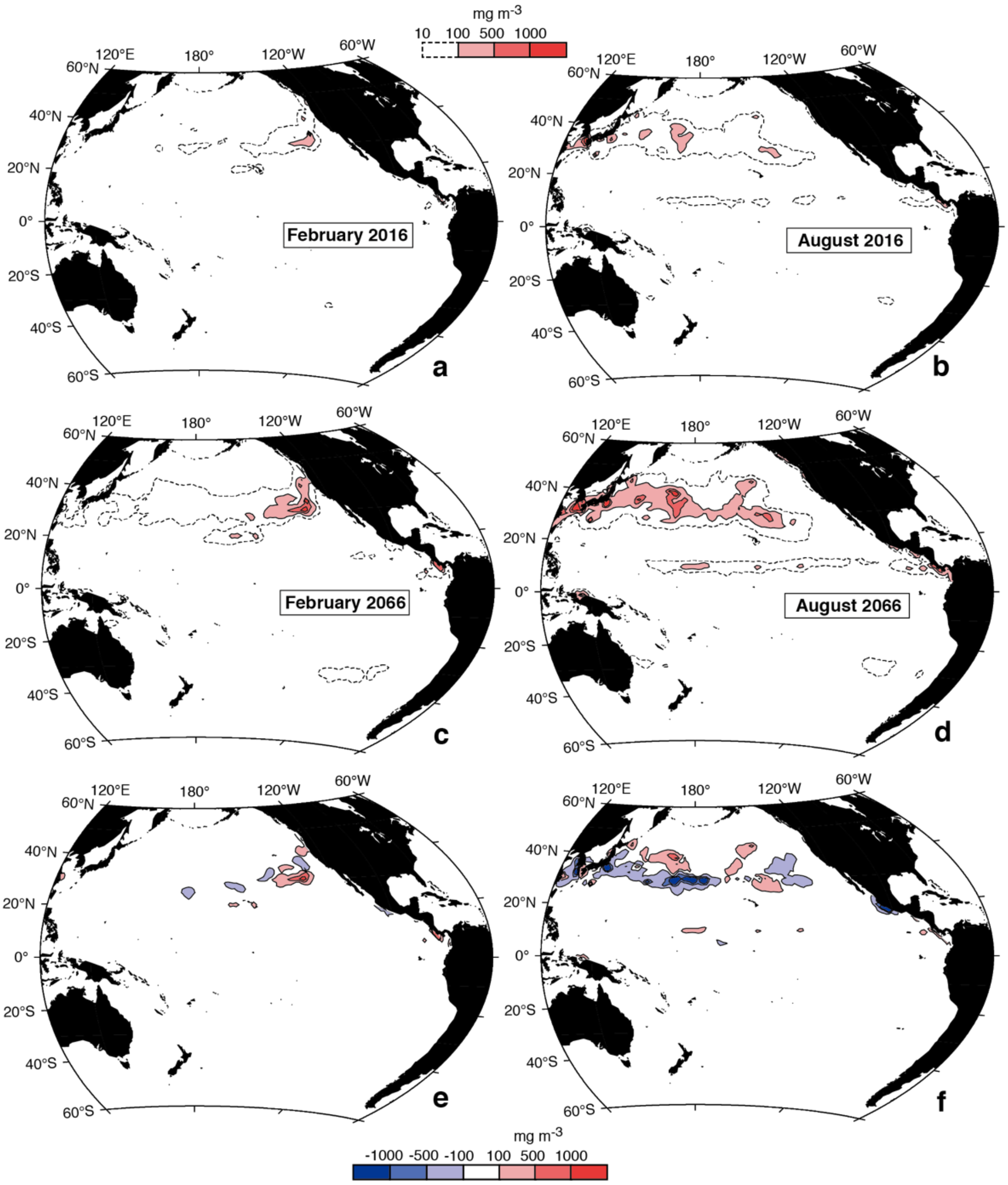

Supplementary Fig. 5. Abundance of microplastics in the present and future. The particle-tracking model repeatedly used ocean currents and Stokes drift in 2010 (2010-comptation). The weight concentrations at the sea surface were averaged in February (a) and August (b) in 2016, and February (c) and August (d) in 2066. The concentrations are shown by a red stippling in the line with the scale in the upper of the figure. The broken curves denote a weight concentration of 10 mg m^{-3}. The difference from 2066 map in the model using ocean currents and Stokes drift in 2015 (2015-computation; Fig. 6) are shown in February (e) and August (f). The difference was computed as the 2010-computation minus the 2015-computation.

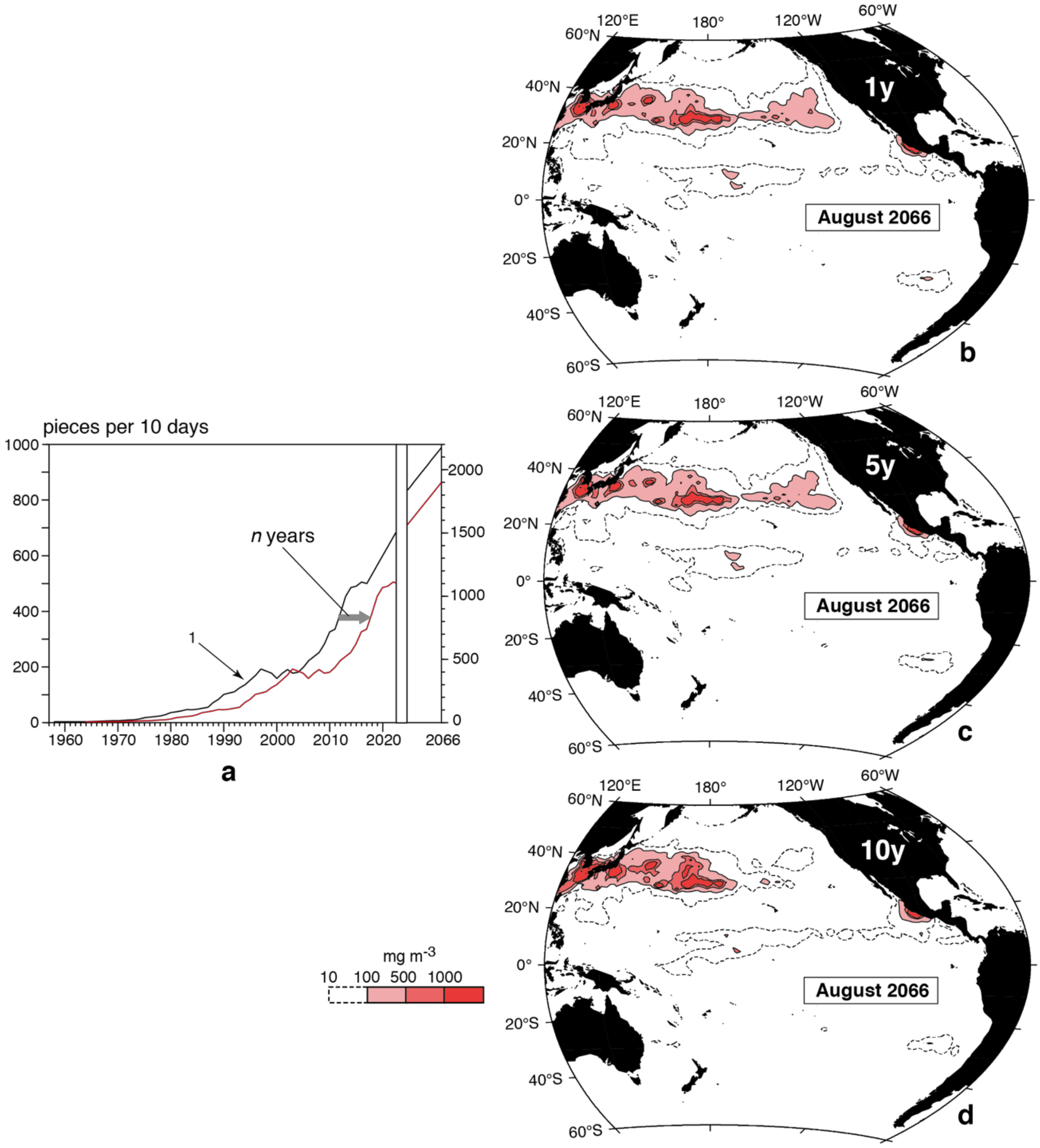

Supplementary Fig. 6. Abundance of microplastics in the present and future. The weight concentrations at the sea surface in August 2066 were computed in models with emission delayed by 1 (b), 5 (c), and 10 (d) years. The particle release in the emission delayed by *n* years is schematically shown for the source 1 (a). According to the root-mean-square errors in Supplementary Table 5, the averaged transit time of 3 (1) years was chosen for the computation to depict the panel b, c (d).

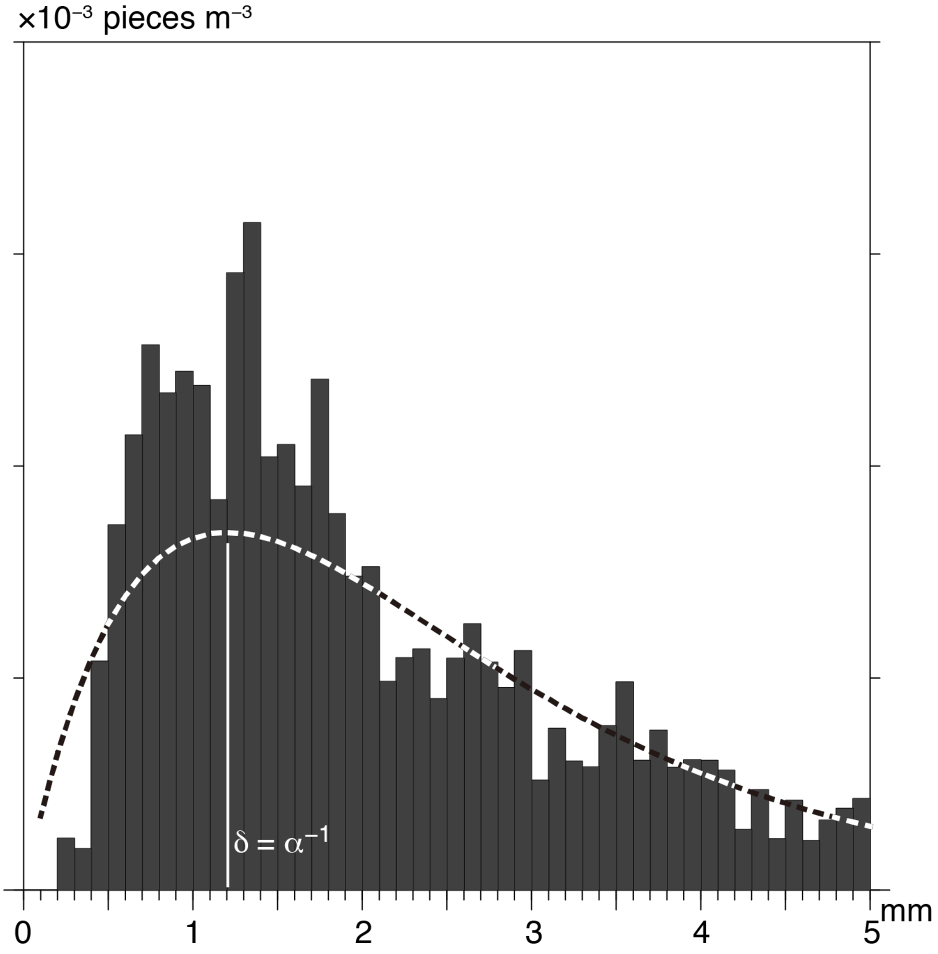

Supplementary Fig. 7. Size distribution of microplastics. The concentrations of microplastics collected along the entire meridional transect in 2016 are shown by the bars in each 0.1-mm size category. The broken curve approximates the observed size distribution using Eq. (4) in a least square sense. The reciprocal of δ in Eq. (4) denotes the mode of the sizes (δ), which was 1.2 mm, as shown in the figure by the white line.

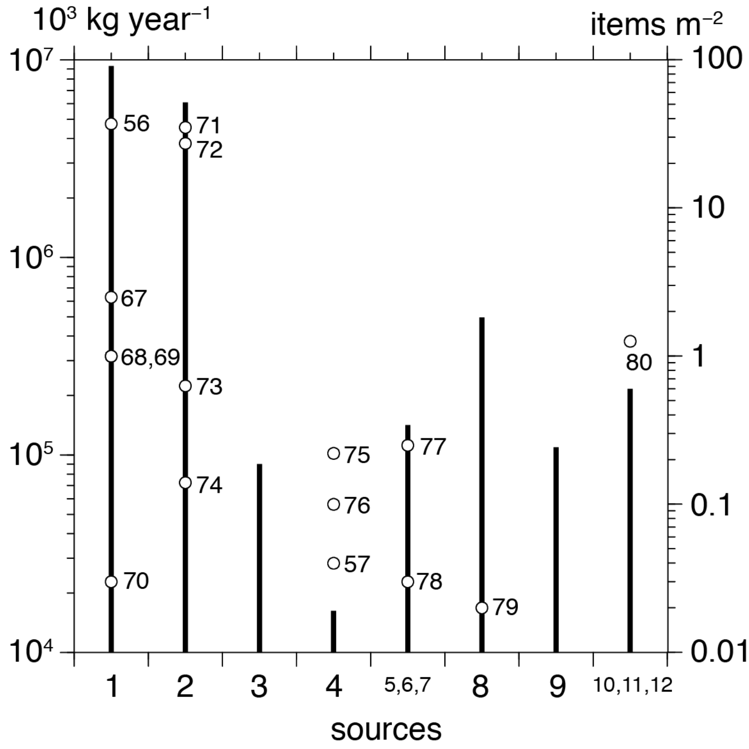

Supplementary Fig. 8. Mismanaged plastic wastes and beach litter in each region. The mismanaged plastic waste[23] (bar; left ordinate) is compared with abundance of macroplastics littered on beaches (open circle with numbers; right ordinate) around sources 1 to 12. Numbers beside open circles indicate articles listed in Supplementary References.

Supplementary Table 1. Field microplastic surveys used in this study

Authors	Period	Region
present study	January−March 2016	western Pacific, and Southern Ocean
Reisser et al. (2013)[22)]	June 2011−August 2012	western South Pacific
Goldstein et al. (2012)[3)]	1972−1987, 1999−2010*	eastern North Pacific

* Months were not shown in a part of surveys included in Goldstein et al. (2012)[3)].

Supplementary Table 2. Modeled emission of microplastics. Mismanaged plastic waste[23] in the countries below were summed at each source, and particle counts released at the sources in 2016 were proportional to these mismanaged plastic wastes; the minimal number was set to 1 (source 4; Oceania). The particle counts for releases in 2066 were determined by considering an increase in the rate of mismanaged plastic wastes from 2010 to 2025[23], which was linearly extrapolated to produce a particle release from 2016 to 2066 under the assumption that there will be no regulation/operation to reduce mismanaged plastic waste.

Sources in Fig. 2	Countries included	Mismanaged plastic waste [10^3 kg/year]	Particles released every 10 days in 2016	Particles released every 10 days in 2066
1	China, Japan, N.Korea, S.Korea,	9,300,913	500	2,175
2	Brunei, Cambodia, Indonesia**, Malaysia*, Philippines, Singapore*, Taiwan, Thailand, Vietnam	6,096,573	330	1,864
3	Papua New Guinea	89,835	5	32
4	Australia*, New Zealand	16,231	1	3
5, 6, 7	Canada*, United States*	141,692	4, 3, 3	6, 5, 5
8	Colombia, Costa Rica, El Salvador, Guatemala, Mexico*, Nicaragua*, Panama*	496,839	30	120
9	Ecuador	109,383	5	20
10, 11, 12	Chile, Peru	216,207	4, 3, 3	16, 12, 12
total		16,467,673***	891	4,270

* Unless large cities were concentrated on the Pacific coast (e.g., Costa Rica), the mismanaged plastic wastes were multiplied by a factor of 0.5, approximately the ratio between the length of the coastline facing the Pacific Ocean to that of the total length of coastline for each country.

** multiplied by 0.25

*** This estimate accounts for approximately 52% of the total wastes in the world (31,865,274 tons/year)[23].

Supplementary Table 3. Root mean square error of the meridional slope (f) and decadal variation (g) of microplastic abundance in comparison with observed values. Errors in the model with (right) and without (left) fishery-based sources are shown.

τ (years)	$\sqrt{(\overline{f}-1)^2+(\overline{g}-1)^2}$	
	Without fishery-based sources	With fishery-based sources
∞	9.6	12.0
10	4.2	5.2
5	1.9	2.4
3	0.47	0.79
1	1.0	0.95

$\overline{f} = f_\tau/f_{obs}$, where f_τ (f_{obs}) is the slope computed in the model with τ (observed slope) in Fig. 5a. $\overline{g} = g_\tau/g_{obs}$, where g_τ (g_{obs}) is the difference between weight concentrations averaged in 1990–2010 and those averaged in 1972–1987 computed in the model with τ (observed) in Fig. 5b.

Supplementary Table 4. Modeled fishery-derived microplastics emissions.

Sources in Fig. 2	Countries included	Ratio of fish catches* (%)	Suspected plastic waste from fishery (10^3 kg/year)	Particles released every 10 days in 2016	Particles released every 10 days in 2066
1	China, Japan, N.Korea, S.Korea,	46.8	1,544,400	85	370
2	Brunei, Cambodia, Indonesia, Malaysia, Philippines, Singapore, Taiwan, Thailand, Vietnam	23.9	788,700	43	242
3	Papua New Guinea	0.5	16,500	1	6
4	Australia, New Zealand	1.6	52,800	3	10
5, 6, 7	Canada, United States	6.3	207,900	6, 4, 4	9,7,7
8	Colombia, Costa Rica, El Salvador, Guatemala, Mexico, Nicaragua, Panama	3.6	118,800	7	30
9	Ecuador	0.9	29,700	1	5
10, 11, 12	Chile, Peru	16.4	541,200	10,8,8	40,30,30
total		100	3,300,000	180	786

* Fish catches in neighboring seas in the Pacific Ocean[66] were used for the computation. For example, the fish catches from area 61 (western North Pacific and marginal seas) were used as source 1 because most fish catches were included there (e.g., 90% in Japan, 97% in China). See FAO yearbook[66] for numbering of areas.

Supplementary Table 5. Root mean square error of the meridional slope and decadal variation of microplastic abundance in comparison with observed values for cases with time intervals taken for the fragmentation in the emission model

		time interval (years)		
		1	5	10
τ (years)	∞	10.3	8.6	13.8
	10	4.5	3.8	7.0
	5	2.1	1.9	3.8
	3	0.6	0.6	1.4
	1	1.1	1.1	0.8

Supplementary Table 6. Classes, species, particle diameters, and weight concentrations for laboratory-based studies compared to the present model prediction. Exposure time, polymer types, tested concentrations, and effects in each experiment are described in Table S1 of de Sá (2018)[48]. The diameters and minimal weight concentrations found to be harmful to organism in the experiments are shown. When a concentration was reported in particles per unit water volume in a laboratory-based study, weight concentration was computed assuming a spherical shape, a given diameter (listed below), and a specific weight of 1. The laboratory-based studies used for this purpose are listed in Supplementary References (see Ref. numbers below).

Class	Species	Particle diameter in Fig. 9 (μm)	Weight concentration in Fig. 9 (mg m^{-3})	Ref.
Echinodermata	*Paracentrotus lividus*	0.05	2,610†	81
		6	113**	18
		40*††	5,000	
	Tripneustes gratilla	27.5*	3,266**	82
Rotifera	*Brachionus koreanus*	0.05, 0.5	10^4	83
Mollusca	*Crassostrea gigas*	4*	23	84
	Mytilus edulis	40*††	2.5×10^6	15
		0.03	10^5	81
	Mytilus galloprovincialis	0.05	1,000	86
		50*	2×10^7	87
		25*††	1.3×10^5**	19
	Scrobicularia plana	20	1,000	88
Crustacea	*Hyalella azteca*	18.5*	3,315**	89
		47.5*	2,525**	
	Tigriopus japonicus	0.05	1,250	90
		0.5	2.5×10^4	
	Centropages typicus	7.3	814**	91

Supplementary Table 6. (continued)

Crustacea	*Palaemonetes pugio*	59††	5,376**	20
		75††	1.1×10^{4}**	
		83††	1.5×10^{4}**	
		116††	4.1×10^{4}**	
		165††	1.2×10^{5}**	
	Artemia franciscana	0.1	1,000	92
	Daphnia galeata	0.05	5,000	93
	Daphnia magna	0.07	220	94
		0.11*	10^{6}	95
		731*	1.25×10^{4}	96
		0.2	25,960†	97
		0.1	10^{4}	98
		3*	1,413**	99
		1††	1.25×10^{4}	100
		0.1	1,000	101
		0.052	7.5×10^{4}	49
	Calanus helgolandicus	20††	330**	16
	Paracyclopina nana	0.05	1	102
		0.5	10	
	Parvocalanus crassirostris	7.5	4,418**	103
Fish	*Carassius carassius*	0.053	10^{5}	49
	Pomatoschistus microps	3*	18.4	104
		460*	5,097**	105
		3	2,160	106

Supplementary Table 6. (continued)

	Clarias gariepinus	30*	50	107
Fish	*Danio rerio*	5	20	50
		0.07	2,000	
		8.8*	500	108
		0.05	1,000	109
		0.45	1,000	
		0.05	1,000	110

* Median of particle sizes used in the experiment

** Weight concentration converted from particle count per unit water volume

† The concentration was not the lower limit, but the median effective concentration (EC50) provided by each study.

†† Plastic beads free of additives were used.

Supplementary References

66. Food and Agriculture Organization of United Nations FAO yearbook. Fishery and Aquaculture Statistics. 2015, 78pp (2017)
67. Kusui, T. & Noda, M. International survey on the distribution of stranded and buried litter on beaches along the Sea of Japan. *Mar. Pollut. Bull.*, **47**, 175-179 (2003).
68. Lee, J. et al. Distribution and Size Relationships of Plastic Marine Debris Relationships of Plastic Marine Debris. *Arch. Environ. Contam. Toxicol.* **69**, 288–298 (2015).
69. Lee., J. et al. Relationships among the abundances of plastic debris in different size classes on beaches in South Korea. *Mar. Pollut. Bull.*, **77**, 349-354 (2013).
70. Zhou et al. Assessment of marine debris in beaches or seawaters around the China Seas and coastal provinces. *Waste Management*, **48**, 652-660 (2016).
71. Zhao, S., Zhu, L. & Li, D. Characterization of small plastic debris on tourism beaches around the South China Sea. *Reg. Stud. Mar. Sci.*, **1**, 55-62 (2015).
72. Syakti, A. D. et al. Beach macro-litter monitoring and floating microplastic in a coastal area of Indonesia. *Mar. Pollut. Bull.*, **122**, 217-225 (2017).
73. Liu, T.-K., Wang, M.-W. & Chen, P. Influence of waste management policy on the characteristics of beach litter in Kaohsiung, Taiwan. *Mar. Pollut. Bull.*, **72**, 99-106 (2013).
74. Kuo, F.-J. & Huang, H.-W. Strategy for mitigation of marine debris: Analysis of sources and composition of marine debris in northern Taiwan. *Mar. Pollut. Bull.*, **83**, 70-78 (2014).
75. Smith, S. D. A. & Markic, A. Estimates of Marine Debris Accumulation on Beaches Are Strongly Affected by the Temporal Scale of Sampling. *PLoS One* **8**, e83694. DOI:10.1371/journal.pone.0083694 (2013).
76. Cunningham, D. J. & Wilson, S. P. Marine debris on beaches of the greater Sydney region. *J. Coast. Res.*, **19**, 421-430 (2003).
77. Silva-Iñiguez, L & Fischer, D. W. Quantification and classification of marine litter on the municipal beach of Ensenada, Baja California, Mexico. *Mar. Pollut. Bull.*, **46**, 132-138 (2003).
78. Kataoka, T., Murray, C. C. & Isobe, A. Quantification of marine macro-debris abundance around Vancouver Island, Canada, based on archived aerial photographs processed by projective transformation. *Mar. Pollut. Bull.*, **132**, 44-51 (2018).
79. Gracia C., A., Rangel-Buitrago, N. & Flórez, P. Beach litter and woody-debris colonizers on the Atlantico department Caribbean coastline, Colombia. *Mar. Pollut. Bull.*, **128**, 185-196

(2018).

80. Bravo, M. et al. Anthropogenic debris on beaches in the SE Pacific (Chile): Results from a national survey supported by volunteers. *Mar. Pollut. Bull.*, **58**, 1718-1726 (2009).
81. Della Torre, C. et al. Accumulation and embryotoxicity of polystyrene nanoparticles at early stage of development of sea urchin embryos *Paracentrotus lividus*. *Environ. Sci. Technol.* **48**, 12302-12311 (2014).
82. Kaposi, K. L., Mos, B., Kelaher, B.P. & Dworjanyn, S.A., Ingestion of microplastic has limited impact on marine larva. *Environ. Sci. Technol.* **48**, 1638-1645 (2014).
83. Jeong, C.B. et al. Microplastic size-dependent toxicity, oxidative stress induction and and p-JNK and p-p38 Activation in the Monogonont Rotifer (*Brachionus koreanus*). *Environ. Sci. Technol.* **50**, 8849-8857 (2016).
84. Sussarellu R. et al. Oyster reproduction is affected by exposure to polystyrene microplastics. *Proc. Natl. Acad. Sci. USA* **113**, 2430-2435 (2016).
85. Wegner, A., Besseling, E., Foekema, E.M., Kamermans, P. & Koelmens, A.A. Effects of nanopolystyrene on the feeding behavior of the blue mussel (*Mytilus edulis* L.). *Environ. Toxicol. Chem.* **31**, 2490-2497 (2012).
86. Canesi, L. et al. Evidence for immunomodulation and apoptotic processes induced by cationic polystyrene nanoparticles in the hemocytes of the marine bivalve *Mytilus*. *Mar. Environ. Res.* **111**, 34-40 (2015).
87. Avio, C.G. et al. Pollutants bioavailability and toxicological risk from microplastics to marine mussels. *Enviro. Pollut.* **198**, 211-222 (2015).
88. Ribeiro, F. et al. Microplastics effects *Scrobicularia plana*. *Mar. Pollut. Bull.* **122**, 379-391 (2017).
89. Au, S. Y., Bruce, T. F., Bridges, W. C. & Klaine, S. J. Responses of *Hyalella azteca* to acute and chronic microplastic exposure. *Environ. Toxicol. Chem.* **34**, 2564-2572 (2015).
90. Lee, K.W., Shim, W.J., Kwon, O.Y. & Kang, J.H., 2013. Size-dependent effects of micro polystyrene particles in the marine copepod *Tigriopus japonicas*. *Environ. Sci. Technol.* **47**, 11278-11283 (2013).
91. Cole, M. et al. Microplastic ingestion by zooplankton, *Environ. Sci. Technol.* **47**, 6646–6655 (2013).
92. Gambardella, C. et al. Effects of polystyrene microbeads in marine planktonic crustaceans. *Ecotoxicol. Environ. Saf.* **145**, 250-257 (2017).
93. Cui, R., Kim, S. W. & An, Y. J. Polystyrene nanoplastics inhibit reproduction and induce

abnormal embryonic development in the freshwater crustacean *Daphnia galeata*. *Sci. Rep.* **7**, 12095. DOI: 10.1038/s41598-017-12299-2 (2017).

94. Besseling, E., Wang, B., Lurling, M. & Koelmans, A. A. Nanoplastic Affects Growth of S. obliquus and Reproduction of *D. magna*. *Environ. Sci. Technol.* **48**, 12336-12343 (2014).
95. Booth, A. M., Hansen, B. H., Frenzel, M., Johnsen, H. & Altin, D. Uptake and toxicity of methylmethacrylate-based nanoplastic particles in aquatic organisms. *Environ. Toxicol. Chem.* **35**, 1641-1649 (2016).
96. Jemec, A., Horvat, P., Kunej, U., Bele, M. & Krzan, A. Uptake and effects of microplastic textile fibers on freshwater crustacean *Daphnia magna*. *Envron. Pollut.* **219**, 201-209 (2016).
97. Kim, D., Chae, Y. & An, Y. J. Mixture Toxicity of Nickel and Microplastics with Different Functional Groups on *Daphnia magna*. *Environ. Sci. Technol.* **51**, 12852-12858 (2017).
98. Nasser, F. & Lynch, I. Secreted protein eco-corona mediates uptake and impacts of polystyrene nanoparticles on *Daphnia magna*. *J. Proteom.* **137**, 45-51 (2016).
99. Ogonowski, M., Schur, C., Jarsen A. & Gorokhova, E. The Effects of Natural and Anthropogenic Microparticles on Individual Fitness in *Daphnia magna*. *PLoS One* **11**, e0155063. DOI: 10.1371/journal.pone.0155063 (2016).
100. Rehse, S., Kloas, W. & Zarfl, C. Short-term exposure with high concentrations of pristine microplastic particles leads to immobilisation of *Daphnia magna*. *Chemosphere* **153**, 91-99 (2016).
101. Rist, S., Baun, A. & Hartmann, N. B. Ingestion of micro- and nanoplastics in *Daphnia magna* – Quantification of body burdens and assessment of feeding rates and reproduction. *Environ. Pollut.* **228**, 398-407 (2017).
102. Jeong, C.-B. et al. Adverse effects of microplastics and oxidative stress-induced MAPK/Nrf2 pathway-mediated defense mechanisms in the marine copepod *Paracyclopina nana*. *Sci. Rep.* **7**, 41323. DOI: 10.1038/srep41323 (2017).
103. Heinder, F. M. et al. Toxic effects of polyethylene terephthalate microparticles and Di(2-ethylhexyl)phthalate on the calanoid copepod, *Parvocalanus crassirostris*. *Ecotoxicol. Environ. Saf.* **141**, 298-305 (2017).
104. Oliveira, M., Ribeiro, A., Hylland, K. & Guilhermino, L. Single and combined effects of microplastics and pyrene on juveniles (0^+ group) of the common goby *Pomatoschistus microps* (teleostei: gobiidae). *Ecol. Indic.* **34**, 641-647 (2013).
105. de Sá, L. C., Luís, L. G. & Guilhermino, L. Effects of microplastics on juveniles of the common goby (*Pomatoschistus microps*): confusion with prey, reduction of the predatory

performance and efficiency, and possible influence of developmental conditions. *Enviro. Pollut.* **196**, 359- 362 (2015).

106. Luis, L., Ferreira, P., Fonte, E., Oliveira, M. & Guilhermino, L. Does the presence of microplastics influence the acute toxicity of chromium(VI) to early juveniles of the common goby (*Pomatoschistus microps*)? A study with juveniles from two wild estuarine populations. *Aquat. Toxicol.* **164**, 163-174 (2015).
107. Karami, A., Romano, N., Galloway, T. & Hamzah, H. Virgin microplastics cause toxicity and modulate the impacts of phenanthrene on biomarker responses in African catfish (*Clarias gariepinus*). *Environ. Res.* **151**, 58-70 (2016).
108. Karami, A., Groman, D. B., Wilson, S. P., Ismail, P. & Neela, V. K. Biomarker responses in zebrafish (*Danio rerio*) larvae exposed to pristine low-density polyethylene fragments. *Environ. Pollut.* **223**, 466-475 (2017).
109. Chen, Q. et al. Quantitative investigation of the mechanisms of microplastics and nanoplastics toward zebrafish larvae locomotor activity. *Sci. Total Environ.* **584-585**, 1022-1031 (2017).
110. Chen, Q. et al. Enhanced uptake of BPA in the presence of nanoplastics can lead to neurotoxic effects in adult zebrafish. *Sci. Total Environ.* **609**, 1312-1321 (2017).
